# Plasma 25-hydroxyvitamin D concentration and risk of type 2 diabetes and pre-diabetes: 12-year cohort study

**DOI:** 10.1371/journal.pone.0193070

**Published:** 2018-04-19

**Authors:** Sue K. Park, Cedric F. Garland, Edward D. Gorham, Luke BuDoff, Elizabeth Barrett-Connor

**Affiliations:** 1 Department of Preventive Medicine, Seoul National University College of Medicine, Jongno-gu, Seoul, Korea; 2 Department of Biomedical Science, Seoul National University Graduate School, Jongno-gu, Seoul, Korea; 3 Cancer Research Institute, Seoul National University, Jongno-gu, Seoul, Korea; 4 Department of Family Medicine and Public Health, University of California, San Diego, La Jolla, California, United States of America; University of Queensland, AUSTRALIA

## Abstract

**Background:**

It has been reported that higher plasma 25-hydroxyvitamin D is associated with lower risk of type 2 diabetes. However the results to date have been mixed and no adequate data based on a cohort are available for the high end of the normal range, above approximately 32 ng/ml or 80 nmol/L.

**Methods:**

We performed a cohort study of 903 adults who were known to be free of diabetes or pre-diabetes during a 1997–1999 visit to a NIH Lipid Research Centers clinic. Plasma 25(OH)D was measured at Visit 8 in 1977–1979. The mean age was 74 years. The visit also included fasting plasma glucose and oral glucose tolerance testing.

Follow-up continued through 2009.

**Results:**

There were 47 cases of diabetes and 337 cases of pre-diabetes. Higher 25(OH)D concentrations (> 30 ng/ml) were associated with lower hazard ratios (HR) for diabetes: 30–39 ng/ml or 75–98 nmol/L: HR = 0.31, 95% CI = 0.14–0.70; for 40–49 ng/ml or 100–122 nmol/L: HR = 0.29, CI = 0.12–0.68; for > 50 ng/ml or 125 nmol/L: HR = 0.19, CI = 0.06–0.56. All HRs are compared to < 30 ng/ml or 75 nmol/L. There was an inverse dose-response gradient between 25(OH)D concentration and risk of diabetes with a *p* for trend of 0.005. Each 10 ng/mL or 25 nmol/L higher 25(OH)D concentration was associated with a HR of 0.64, CI = 0.48–0.86. 25(OH)D concentrations were more weakly inversely associated with pre-diabetes risk, and the trend was not significant.

**Conclusion:**

Further research is needed on whether high 25(OH)D might prevent type 2 diabetes or transition of prediabetes to diabetes.

## Introduction

The public health impact of vitamin D deficiency has received attention due to the discovery of associations between low plasma concentrations of vitamin D metabolites and higher risk of several cancers, cardiovascular disease, bone fractures [1–3] and the metabolic syndrome [4]. Trends in energy intake and anthropometric characteristics have paralleled the increase in incidence of type 2 diabetes mellitus. It is unclear whether vitamin D deficiency might be contributing to increased risk [5].

If defining 25(OH)D levels < 32 ng/ml (< 80 nmol/L) as deficiency [6], 77% of U.S. adults were deficient. The prevalence of vitamin D deficiency by this criterion has doubled since 1980 in U.S. adults [6].

Several cohort studies have examined the association of circulating 25(OH)D concentrations with risk of diabetes. Of these, 12 found significantly higher incidence rates in individuals with lower circulating 25(OH)D concentrations [5, 7–16]. The association in one was limited to overweight subjects [16]; and the significant finding in another [14] was limited to women. One study found a benefit of 25(OH)D ≥ 11 ng/ml compared to < 11 ng/ml, but no further benefit with higher concentrations [15]. Two studies reported a significant inverse association in men, but not women [17, 18]. One study reported a favorable association that did not reach statistical significance [19]. A study by Schafer et al. reported a statistically significant inverse association between 25(OH)D and hazard ratio of diabetes after adjustment for age and clinic location, but that was weak and no longer statistically significant after adjustment for more factors that included BMI [20].

The association of plasma 25(OH)D deficiency with risk of diabetes also has been examined in four meta-analyses [11, 13, 21, 22], and all reported an inverse association of circulating 25(OH)D with risk of diabetes.

The aim of this study was to examine whether lower concentrations of 25(OH)D or 1,25(OH)_2_D were associated with higher incidence of diabetes and pre-diabetes in a prospective cohort study with an overall follow-up period of 12 years.

This cohort may have a lower than usual prevalence of vitamin D deficiency due to year-round sunshine and good weather in a sunny and clear area of southern California [23]. It may also be possible that the cohort has a lower than usual prevalence of vitamin D deficiency due to a higher standard of education and socioeconomic status and a high proportion of Caucasians. This cohort has the highest known published median 25(OH)D concentration, 42 ng/ml or 105 nmol/L in men [24] and 39 ng/ml or 98 nmol/L in women [25] of any population that has reported data on diabetes incidence by 25(OH)D. No previous study of the association of 25(OH)D with diabetes has included a substantial population in the high range of > 30 ng/ml or 75 nmol/L.

## Methods

### Participants

Participants were from the Rancho Bernardo Study, a population-based cohort of primarily older, middle-income, community-dwelling Caucasian adults living in a southern California suburb. They were subjects in a Lipid Research Clinics Prevalence Study consisting of a series of visits. This was part of an NIH study of lipid-lowering agents established in 1972 [26]. The individuals did not receive any medication, but rather served solely as an untreated comparison group. From 1997 to 1999, 1,098 surviving community-dwelling participants attended a follow-up visit known as Visit 8. Of these, 1,080 received measurements of their plasma 25(OH)D. Details of the inclusions are shown in the Supplementary Figure.

We followed the cases until diagnosis of pre-diabetes or diabetes and non-cases until their last test of 8-hour fasting plasma glucose (8-FPG) and oral glucose tolerance testing (OGTT). Of the total participants, 52 had a history of diabetes and were excluded at baseline. Of the remaining *N* = 1,028 participants, we first screened for diabetes using 8-FPG and excluded 60 participants with 8-FPG concentrations ≥ 126 mg/dL or 7.0 mmol/L or had missing data on 8-FPG. We further excluded 65 with 2-hour OGTT > 200 mg/dL or 11.1 mmol/L.

Finally, a total of 903 participants were included in this study. Of these, 47 incident type 2 diabetes and 337 pre-diabetes cases were ascertained during 1997–2009. The multivariate analyses included 46 diabetes cases and 337 pre-diabetes cases. The one fewer diabetes case was due to missing data on covariates on one individual.

All willing participants were followed and are presently being followed, including the diabetes and pre-diabetes cases. The follow-up rate through 2009 was 87%.

A flowchart in S1 Fig shows that no individual who was diabetic or pre-diabetic at baseline in 1977–1979 was allowed to enter the cohort of *N* = 903 individuals who constituted this study. As a result, the cohort was, to the standard of technology in 1977–1979 and WHO definitions of diabetes and pre-diabetes in international use, free of diabetes or pre-diabetes.

Individuals who developed diabetes during follow up were counted as incident cases to determine the hazard rate. If an individual developed only pre-diabetes, he or she was counted as a case of pre-diabetes, unless they later developed diabetes. If so, they were counted once, as a case of diabetes, to avoid counting any individual more than once. The University of California, San Diego Human Subjects Protections Program approved this study, and all participants gave written informed consent.

### Data collection

During the 1997–1999 visit, participants completed standardized questionnaires that inquired about myocardial infarction, stroke, angina pectoris, and peripheral claudication, current medications, cigarette smoking, alcohol consumption, and physical exercise. Height and weight were measured using a Lipid Research Clinics calibrated stadiometer and balance-beam scale. Systolic and diastolic blood pressures were measured twice in seated subjects after a 5-minute rest period, using the standard Hypertension Detection and Follow-up Program protocol [27]. Body mass index was calculated as weight in kilograms / height in meters^2^. Use of vitamin D and calcium supplements at baseline was determined using a questionnaire.

The primary exposure variables were plasma concentrations of 25(OH)D and 1,25(OH)_2_D. Blood was obtained by venipuncture, after an overnight fast, and tubes were protected from sunlight. Plasma was separated and stored at −70°C within 30 minutes of collection. Plasma 25(OH)D and 1,25(OH)_2_D concentrations were measured in the Holick-Chen Laboratory at Boston University using vitamin D competitive binding protein recognition and chemiluminescence detection (Stillwater MN, USA:Diasorin) [28]. To convert 25(OH)D from nanograms per milliliter to nanomoles per Liter, multiply nanograms/Liter times 2.5 [29].

The intra- and inter-assay coefficients of variation for the assay were 8% and 10%, respectively [28] the limit of detection was 5 ng/mL or 13 nmol/L, and the reference range was 10–52 ng/mL or 25–130 nmol/L. For 1,25(OH)_2_D, the intra- and inter-assay coefficients of variation were 5–10% and 10–15%, respectively; the limit of detection was 4.6 pg/mL or 12 pmol/L [28].

### Case definition

Type 2 diabetes cases were defined by World Health Organization criteria of 1999 as a ≥ 8-hour FPG, or 8-FPG ≥ 126 mg/dL or ≥ 7.0 mmol/L and/or 2-hour oral glucose tolerance test, or 2-OGTT, of > 200 mg/dL or > 11.1 mmol/L. Pre-diabetes was defined as 8-FPG of 100–125 mg/dL, or 5.5–6.9 mmol/L; or 2-OGTT of 140–200 mg/dL, or 7.8–11.1 mmol/L [30].

Blood specimens for 8-FPG were collected every 2 years, in all seasons. If the 8-FPG concentration ever was ≥ 100 mg/dl or 5.5 mmol/L a 2-OGTT was performed. The measurement of 25(OH)D in plasma was performed once, in 1997–1999. The cohort had been assembled earlier, in 1972, and the participants were interviewed and examined, or completed questionnaires, every 2 years. There were 47 incident cases of diabetes and 337 incident cases of pre-diabetes.

### Statistical analysis

Plasma 25(OH)D categories of < 30, 30–39, 40–49 and ≥ 50 ng/ml or < 75, 75–98, 100–122 and ≥ 125 mmol/L are even multiples of 5 ng/ml, and were chosen for this analysis because they are standard and readily understandable. Covariates other than gender were continuous, including BMI, waist circumference, plasma high density lipoprotein and triglyceride concentrations. One covariate, calcium supplementation, was entered as a dichotomous covariate, because further detail was not available. Intake of vitamin D supplements could not be used as a covariate in the regression model since every participant who took a vitamin D supplement also took a calcium supplement.

Chi-square tests for categorical variables and *t*-tests for continuous variables were used to identify differences between participants who developed diabetes or pre-diabetes compared to those who did not. Cox proportional hazards models were used to determine hazard ratios (HRs) and 95% confidence intervals [31] for categories of 25(OH)D and 1,25(OH)_2_D, with adjustment for six covariates, including sex, calcium supplement use, body mass index, waist circumference, plasma high-density lipoprotein cholesterol, and triglyceride concentrations. These were all continuous scales at baseline.

Covariates for multivariate analyses were chosen using backward multivariate logistic regression including all significant variables (*p* < 0.05) with all exposure variables. Waist circumferences and calcium supplementation were selected as significant covariates. Plasma concentrations of 25(OH)D and 1,25(OH)_2_D had skewed distributions, so they were entered in multivariate models as categorical variables. Heterogeneity was evaluated by the Cochran Q test [31].

For a sensitivity analysis of whether the association of plasma 25(OH)D with diabetes was explained by traditional, widely accepted diabetes risk factors, four additional analyses were performed using risk scores for propensity to develop diabetes that were calculated using algorithms developed by the Centers for Disease Control and Prevention based on NHANES-III Third National Health and Nutrition Examination Survey data [32] and ARIC, the Atherosclerosis Risk in Communities study [33].

These scores were used to adjust the hazard rates for diabetes risk factors including age, waist circumference, history of gestational diabetes, family history of diabetes, weight, height, blood pressure, and regular exercise for the NHANES-III risk score [32]; and age, sex, race, hypertension, smoking history, resting pulse, parental history of diabetes, height, weight and waist circumference for the ARIC risk score [33].

Subgroup analyses stratified for the presence of hyperparathyroidism, regular strenuous exercise, metabolic syndrome, and high vs. low diabetes risk score according to the NHANES-III and ARIC algorithms were performed to identify any effect modifiers of the association between vitamin D metabolite concentrations and diabetes risk. All *p*-values were two-tailed. All analyses were conducted using SAS Version 9.2 (SAS Institute, Cary, NC). Anonymized data are in S1 Table.

## Results

Results according to continuous variable at baseline are shown in Table 1. Median follow-up time until diagnosis of diabetes or pre-diabetes was as follows: 4.5 years for diabetes cases; 4.1 years for pre-diabetes cases; and 12.5 years for the total cohort. Range of age of the cohort at baseline was 38–97 years, with a mean of 74 years. Body mass index, waist circumference, fasting plasma glucose, triglyceride concentrations and systolic blood pressure were higher in individuals who became cases of diabetes during the follow-up period than in those who did not, as shown in Table 1.

**Table 1:** Baseline characteristics of diabetes cases, pre-diabetes cases and non-cases in the Rancho Bernardo cohort, 1997–1999.

		Diabetes			Pre-diabetes		
	Cohort (n = 903)	Non-cases (n = 856)	Cases (n = 47)^a^		Non-cases (n = 519)	Cases (n = 349)	
	Mean (SE)	Mean (SE)	Mean (SE)	*p-value*^b^	Mean (SE)	Mean (SE)	*p-value*^b^
Age (years)	74.1 (0.3)	74.2 (0.4)	73.7 (1.2)	0.768	73.7 (0.5)	74.8 (0.5)	0.124
Fasting blood sugar (mg/dl)	99.3 (0.4)	97.9 (0.3)	126.5 (2.8)	<0.001	92.6 (5.2)	106.1 (0.4)	<0.001
BMI (Kg/m^2^)	25.4 (0.1)	25.2 (0.1)	29.1 (0.8)	<0.001	24.4 (0.1)	26.4 (0.2)	<0.001
Waist circumference (cm)	85.1 (0.4)	84.4 (0.4)	97.9 (2.0)	<0.001	81.3 (0.5)	89.6 (0.7)	<0.001
Triglycerides (mg/dl)	121.9 (2.6)	117.3 (2.1)	206.8 (28.8)	0.003	112.1 (2.5)	126.7 (3.5)	<0.001
HDL (mg/dl)	61.1 (0.6)	61.9 (0.6)	45.5 (2.0)	<0.001	64.6 (0.8)	57.3 (1.0)	<0.001
Systolic blood pressure (mmHg)	135.1 (0.7)	134.8 (0.7)	141.2 (3.0)	0.040	134.6 (1.0)	135.3 (1.0)	0.624
Diastolic blood pressure(mmHg)	74.0 (0.3)	73.9 (0.3)	75.5 (1.3)	0.267	73.7 (0.4)	74.4 (0.5)	0.285
25(OH)D (ng/ml)	41.9 (0.5)	42.2 (0.5)	36.9 (1.6)	0.002	42.7 (0.7)	41.4 (0.7)	0.191
(nmol/L)	104.7 (1.2)	105.4 (1.2)	92.3 (3.9)	0.002	106.7 (1.6)	103.4 (1.9)	0.191
1,25(OH)_2_D (pg/ml)	31.7 (0.6)	31.7(0.6)	31.9(0.6)	0.955	32.2(0.8)	30.9(0.9)	0.274
(pmol/L)	76.2 (1.4)	76.1 (1.4)	76.4 (5.2)	0.955	77.4 (1.9)	74.2 (2.2)	0.274
Follow-up (years)^c^	12.5 (3.3)	12.6 (2.7)	4.5 (5.4)	<0.001	10.1 (8.4)	4.5 (1.6)	<0.001

^a.^ These 47 cases included 35 whose diabetes was diagnosed without any prior diagnosis of pre-diabetes. Those with a prior diagnosis of pre-diabetes were not counted as pre-diabetes cases, in order to avoid counting any individual more than once.

^b.^ *P*-values are from *t*-tests for continuous variables and chi-square tests for categorical variables.

^c.^ For follow-up years, values shown are medians and interquartile ranges.

Use of vitamin D and calcium supplements at baseline was lower in individuals who became diabetes cases than in those who did not. Plasma HDL-cholesterol concentration at baseline was lower in individuals who became diabetes or pre-diabetes cases than in those who did not develop diabetes.

Results according to discrete variables at baseline are shown in Table 2. Males constituted 70% of diabetes cases but only 49% of pre-diabetes cases. Alcohol use, smoking, and self-reported regular strenuous exercise were not significantly associated with incidence of diabetes or pre-diabetes, but there was a borderline adverse trend of higher alcohol use by cases of pre-diabetes.

**Table 2:** Baseline characteristics of non-cases, type 2 diabetes mellitus cases and pre-diabetes cases in the Rancho Bernardo cohort, discrete variables, 1997–1999.

		Diabetes			Pre-diabetes		
	Cohort (n = 903)	Non-cases (n = 856)	Cases (n = 47)^a^		Non-cases (n = 519)	Cases (n = 349)	
	N (%)	N (%)	N (%)	*p-value*^b^	N (%)	N (%)	*p-value*^b^
Male	340 (37.7)	307 (35.9)	33 (70.2)	<0.001	146 (28.1)	171 (49.0)	<0.001
Current alcohol drinking	452 (50.1)	427 (49.9)	25 (53.2)	0.659	246 (47.4)	185 (53.0)	0.105
Ever smoking	492 (54.5)	463 (54.1)	29 (61.7)	0.308	272 (52.4)	199 (57.0)	0.181
Regular strenuous exercise	193 (21.4)	182 (21.3)	11 (23.4)	0.727	110 (21.2)	74 (21.2)	0.998
Calcium supplement use	416 (46.1)	401 (46.9)	15 (31.9)	0.045	271 (52.2)	132 (37.9)	<0.001
Vitamin D supplement use	203 (22.5)	199 (23.3)	4 (8.5)	0.018	31 (25.2)	69 (19.8)	0.064

^a.^ These 47 cases included 35 whose diabetes was diagnosed without any prior diagnosis of pre-diabetes. Those with a prior diagnosis of pre-diabetes were not counted as pre-diabetes cases, in order to avoid counting any individual more than once.

^b.^ *P*-values are from chi-squared tests.

Use of calcium supplements at baseline was associated with lower risk of diabetes (*p* < 0.05). There was a borderline trend linking use of vitamin D supplements at baseline with lower incidence of diabetes (*p* = 0.06).

**Fig 1:**
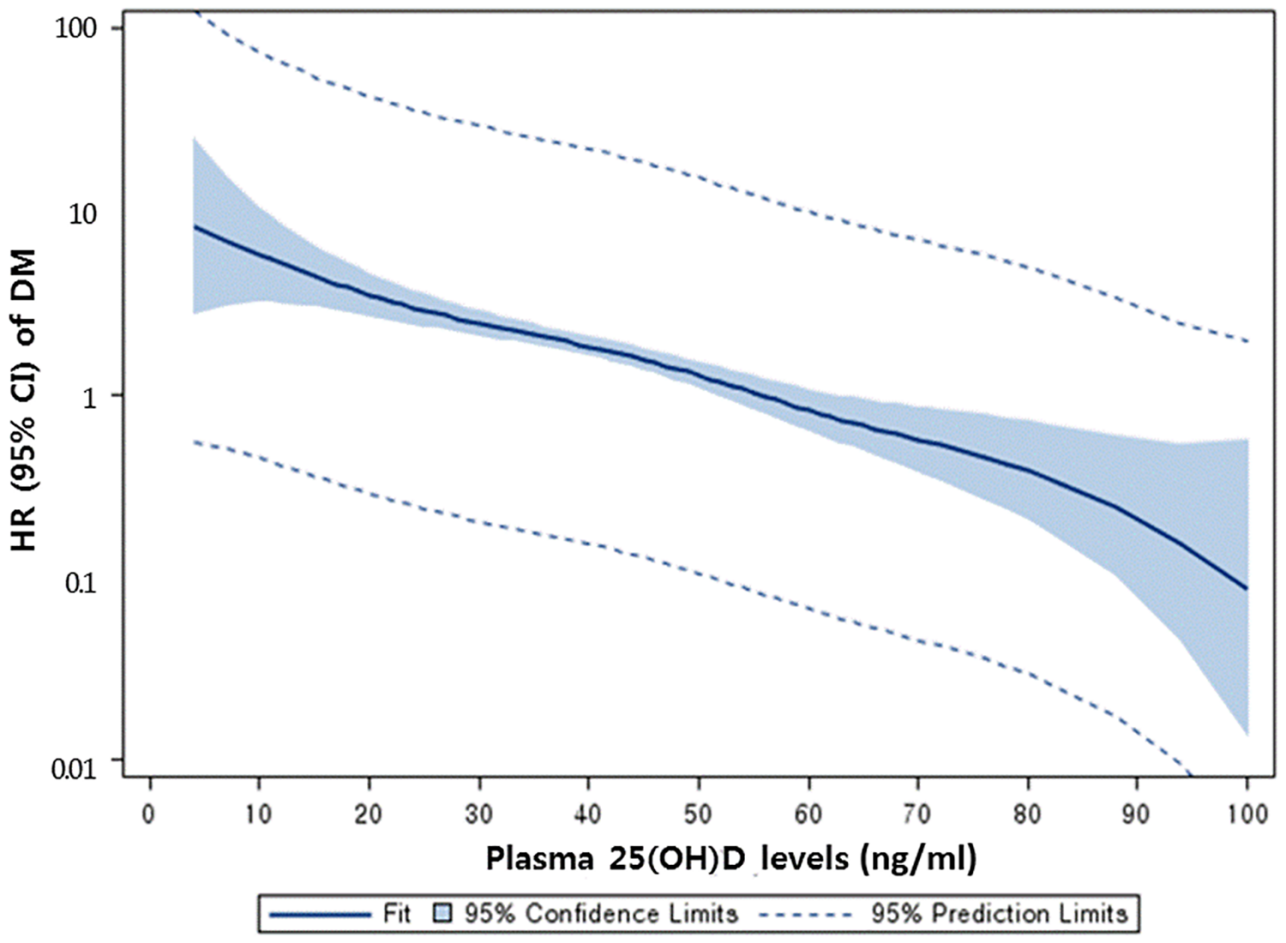
Hazard ratio for type 2 diabetes according to plasma 25(OH)D concentration at baseline, Rancho Bernardo CA, 1997–2009.

As shown in Table 3, a plasma 25(OH)D concentrations > 30 ng/ml or 75 nmol/L was associated with approximately 70% lower incidence of diabetes compared with < 30 ng/ml or 75 nmol/L at baseline. A concentration of 30–39 ng/ml or 75–99 nmol/L was associated with HR = 0.31 and 40–49 ng/ml or 100–124 nmol/L was associated with HR = 0.29 as shown in Fig 1. Hazard ratios were progressively lower in each stratum from the lowest 25(OH)D concentration of ≤ 30 ng/ml or 75 nmol/L to the highest, of ≥ 50 ng/ml or 125 nmol/L. The highest levels of 25(OH)D had an 81% lower incidence rate of diabetes, or HR = 0.19. Each 10 ng/ml or 25 nmol/L higher 25(OH)D was associated with a HR = 0.64.

**Table 3:** Hazard ratios with 95% confidence intervals of type 2 diabetes mellitus (DM) incidence by categories of plasma vitamin D metabolite concentrations in the Rancho Bernardo cohort, 1997–1999.

Plasma metabolite concentration	Person-years of cohort	No. who developed type 2 diabetes (n = 46) ^a^	HR (95% CI) ^b^	Person-years of cohort	No. who developed pre-diabetes (n = 346) ^a^	HR (95% CI) ^b^
25(OH)D (ng/ml) ^c^^,^ ^f^						
< 30	1,331	13	1.00	886	52	1.00
30–39	3,401	13	0.31 (0.14–0.70)	2,621	112	0.75 (0.54–1.05)
40–49	3,465	14	0.29 (0.12–0.68) ^e^	2,529	122	0.88 (0.63–1.24) ^e^
≥ 50	2,199	6	0.19 (0.06–0.56) ^e^	1,728	60	0.66 (0.45–0.97) ^e^
*p*-trend			0.005			0.173
< 30	1,331	13	1.00	886	52	1.00
≥ 30	9,065	33	0.27 (0.13–0.56) ^e^	6,876	294	0.78 (0.57–1.07) ^e^
1,25(OH)_2_D (pg/ml) ^d^^,^ ^g^						
< 20	2,054	7	1.00	1,522	72	1.00
20–29	3,096	20	1.46 (0.61–3.52)	2,237	115	1.01 (0.75–1.36)
30–39	2,657	9	0.94 (0.35–2.57)	1,996	82	0.87 (0.63–1.20)
≥ 40	2,590	10	1.13 (0.42–3.05)	2,010	77	0.81 (0.59–1.13)
*p*-trend			0.781			0.120
< 30	5,150	27	1.00	3,759	187	1.00
≥ 30	5,247	19	0.79 (0.43–1.43)	4,005	159	0.84 (0.68–1.04)

25(OH)D, plasma 25 hydroxyvitamin D; 1,25(OH)_2_D, plasma 1,25 dihydroxyvitamin D

^a.^ 46 diabetes cases and 337 pre-diabetes cases were included in the multivariate analyses, with the difference due to a case of diabetes with missing covariates.

^b.^ Adjusted for sex, body mass index, waist circumference, calcium supplement intake, plasma triglycerides and HDL-cholesterol.

^c.^ 25(OH)D conversions: 30 ng/ml = 75 nmol/L; 40 ng/ml = 100 nmol/L; 50 ng/ml = 125 nmol/L;

^d.^ 1,25(OH)_2_D conversions: 20 pg/ml = 48 pmol/L; 30 pg/ml = 72 pmol/L; 40 pg/ml = 96 pmol/L.

^e.^ There was significant heterogeneity between two hazard ratios on diabetes and pre-diabetes (*P* = 0.025 for ‘40–49 ng/mL’; *P* = 0.039 for ‘≥ 50 ng/mL’; and *P* = 0.009 for ‘30+ ng/mL’)

^f.^ The number of participants in each category of 25-hydroxyvitamin D was 116, 295, 301 and 191, respectively, from lowest to highest category of 25(OH)D.

^g.^ The number of participants in each category of 1,25-dihydroxyvitamin D was 87, 220, 225, 143 and 191, respectively, from lowest to highest category of 1,25(OH)_2_D.

**Fig 2:**
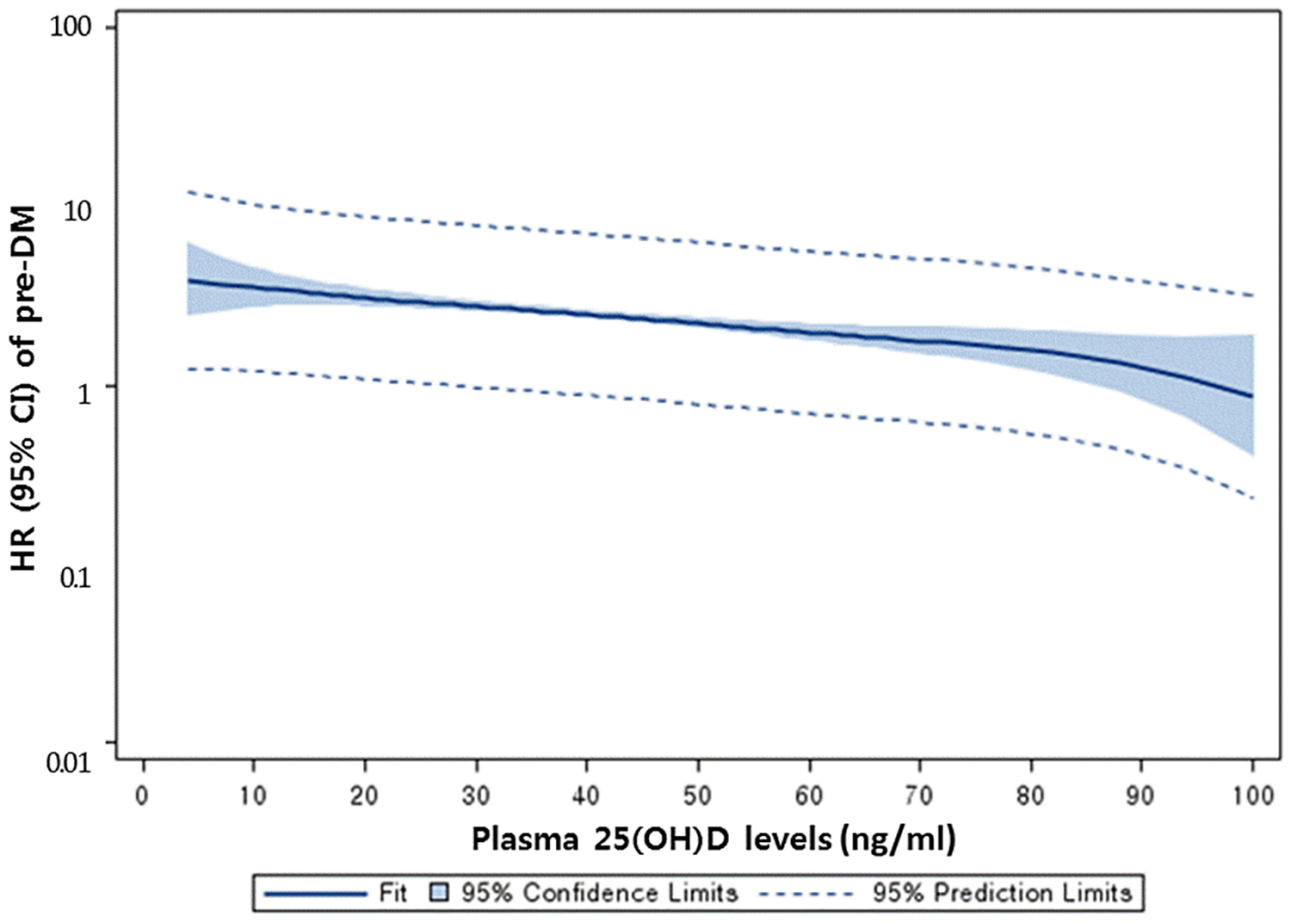
Hazard ratio for pre-diabetes according to plasma 25(OH)D concentration at baseline, Rancho Bernardo CA, 1997–2009.

The association of 25(OH)D with pre-diabetes was weak compared to that with diabetes (Fig 2). For 40–49 ng/ml or 100–124 nmol/L, the *p*-heterogeneity was 0.025 between the two HRs in diabetes and pre-diabetes risk; for > 50 ng/ml or 125 nmol/L, *p*-heterogeneity was 0.039. 25(OH)D concentrations > 50 ng/ml or 125 nmol/L were significantly associated with lower incidence of pre-diabetes. The HR was 0.66.

There were *N* = 241 deaths of participants, leaving *N* = 662 alive through the end of the follow-up period. The mean 25(OH)D concentration in those who died was 38.9 ng/ml, or 97.3 nmol/L. The mean in those who lived was 43.0 ng/ml, or 107.5 nmol/L.

An analysis was performed of the inverse association between serum 25(OH)D and hazard ratios for diabetes according to whether the individual was taking a calcium supplement at baseline, as shown in Table 4. This revealed that the association between 25(OH)D and risk of diabetes may have been slightly stronger in participants who took calcium supplements at baseline. In those who took supplements, there was a hazard ratio of 0.55 with 95% CI of 0.31–0.99 for each 10 ng/ml or 25 nmol/L increase in serum 25(OH)D. By contrast, in participants who took no calcium at baseline, the hazard ratio was 0.69 with 95% CI 0.49–0.98 for each 10 ng/ml or 25 nmol/L increase in serum 25(OH)D. The slightly lower hazard ratio suggests that calcium might enhance the effect of 25(OH)D, but the difference according to calcium supplement use was not statistically significant. The association of plasma 25(OH)D with risk of diabetes persisted after exclusion of individuals taking calcium and/or vitamin D supplements.

**Table 4:** Hazard ratios with 95% confidence intervals of type 2 diabetes incidence by categories of plasma vitamin D metabolite concentrations by use of calcium supplements, Rancho Bernardo cohort, 1997–2009.

Plasma metabolite concentration	Person-years of cohort	No. who developed type 2 DM	HR (95% CI) ^a^	Person-years of cohort	No. who developed PreDM	HR (95% CI) ^a^
25(OH)D (ng/ml)	With calcium supplementation			Without calcium supplementation		
< 30 ng/mL	892	10	1.00	580	36	1.00
30–39	1,920	11	0.37 (0.15–0.96)	1,389	79	0.87 (0.58–1.31)
40–49	1,657	6	0.32 (0.11–0.95) ^b^	1,151	67	0.99 (0.65–1.53) ^b^
≥ 50	1,008	4	0.20 (0.05–0.83) ^b^	743	33	0.71 (0.44–1.17) ^b^
p-trend			0.027			0.398
< 30	892	10	1.00	580	36	1.00
30+	4,585	21	0.33 (0.14–0.78) ^b^	3,283	179	0.88 (0.60–1.28) ^b^
1,25(OH)_2_D (pg/ml)	With calcium supplementation			Without calcium supplementation		
< 30 ng/mL	1,248	13	1.00	815	50	1.00
30–39	2,600	12	0.33 (0.14–0.76)	1,989	90	0.72 (0.50–1.02)
40–49	2,571	11	0.28 (0.11–0.72)	1,853	93	0.88 (0.61–1.27)
≥ 50	1,565	6	0.23 (0.08–0.70) ^c^	1,204	44	0.62 (0.40–0.94) ^c^
p-trend			0.004			0.192
< 30	1,248	13	1.00	815	50	1.00
30+	6,736	29	0.30 (0.14–0.63) ^c^	5,046	227	0.75 (0.54–1.04) ^c^

^a.^ Adjusted for sex, body mass index, waist circumference, calcium supplement intake and blood levels of triglyceride and HDL-cholesterol

^b.^ There was significant heterogeneity between two hazard ratios on diabetes and pre-diabetes (*P* = 0.056 for ‘40–49 ng/mL’; *P* = 0.095 for ‘≥ 50 ng/mL’; *P* = 0.041 for ‘30+ ng/mL’)

^c.^ There was significant heterogeneity between two hazard ratios on diabetes and pre-diabetes (*P* = 0.095 for ‘≥ 40 ng/mL’; *P* = 0.029 for ‘30+ ng/mL’)

Regarding multiple regression analyses of the associations of vitamin D supplements *vs*. calcium supplements, it was not possible to absolutely separate the association of vitamin D supplementation compared to the association with calcium supplementation. This was because all individuals who took vitamin D supplements also took calcium.

As shown in Table 5, the association of low 25(OH)D with high incidence of diabetes persisted after adjustment for NHANES-III and ARIC diabetes risk scores. The association of low 25(OH)D with high risk of diabetes also persisted after stratification for PTH level, regular strenuous exercise, and metabolic syndrome (Table 6).

**Table 5:** Hazard ratios with 95% confidence intervals of type 2 diabetes incidence by categories of plasma 25(OH)D concentration adjusted for different combinations of confounding factors for diabetes, Rancho Bernardo cohort, 1997–2009.

	Risk for diabetes			Risk for pre-diabetes		
Plasma metabolite concentration	HR (95% CI)^b^	HR (95% CI)^c^	HR (95% CI)^d^	HR (95% CI)^b^	HR (95% CI)^c^	HR (95% CI)^d^
25(OH)D (ng/ml) ^a^						
< 30	1.00	1.00	1.00	1.00	1.00	1.00
30–39	0.31 (0.14–0.70)	0.43 (0.20–0.93)	0.44 (0.20–0.95)	0.75 (0.54–1.05)	0.78 (0.56–1.08)	0.78 (0.56–1.08)
40–49	0.29 (0.12–0.68)	0.46 (0.22–0.99)	0.47 (0.22–1.00)	0.88 (0.63–1.24)	0.94 (0.68–1.30)	0.92 (0.66–1.27)
≥ 50	0.19 (0.06–0.56)	0.38 (0.15–0.96)	0.36 (0.14–0.90)	0.66 (0.45–0.97)	0.68 (0.47–0.99)	0.64 (0.45–0.93)

^a.^ 25(OH)D conversions: 30 ng/ml = 75 nmol/L; 40 ng/ml = 100 nmol/L; 50 ng/ml = 125 nmol/L

^b.^ Adjusted for sex, body mass index, waist circumference, calcium supplement intake and blood levels of triglyceride and HDL-cholesterol.

^c.^ Adjusted for the NHANES-III diabetes risk scores. The NHANES-III (the Third National Health and Nutrition Examination Survey) diabetes risk score was developed on the basis of a questionnaire including information on age, waist circumference, gestational diabetes, family history of diabetes, weight and height, blood pressure and exercise (33)

^d.^ Adjusted for the CDC ARIC DM risk score. The CDC ARIC (Atherosclerosis Risk in Communities [ARIC] study in United States Centers for Disease Control and Prevention) DM risk score was based on questionnaire plus blood information such as age, sex, race, hypertension, smoking history, resting pulse, parental history of DM and anthropometric characteristics such as height, waist, and weight from questionnaire and plasma glucose, triglyceride, HDL cholesterol and uric acid in fasting state (32)

**Table 6:** Hazard ratios with 95% confidence intervals of type 2 diabetes mellitus (DM) incidence by plasma 25(OH)D levels after stratification for PTH levels, regular strenuous exercise, metabolic syndrome, and DM risk scores.

Plasma metabolite concentration	Person-years of cohort	No. who developed type 2 DM	HR (95% CI) ^b^	Person-years of cohort	No. who developed type 2 DM	HR (95% CI) ^b^	*P*-heterogeneity ^c^
25(OH)D (ng/ml) ^a^	Normal range of PTH (PTH<60 pg/mL)			Hyperparathyroidism (PTH ≥ 60 pg/mL)			
< 30	814	3	1.00	517	10	1.00	
≥ 30	7,160	27	0.73 (0.21–2.54)	1,905	6	0.06 (0.02–0.25)	0.006
	No regular strenuous exercise			Regular strenuous exercise			
< 30	1,197	11	1.00	143	2	1.00	
≥ 30	6,881	24	0.35 (0.16–0.80)	2,219	9	0.02 (0.002–0.20)	0.046
	No metabolic syndrome			Metabolic syndrome			
< 30	831	3	1.00	509	10	1.00	
≥ 30	6,978	12	0.21 (0.05–0.87)	2,122	22	0.42 (0.17–1.00)	0.419
	Low NHANES-III DM risk scores < Median			High NHANES-III DM risk scores ≥Median			
< 30	654	4	1.00	686	9	1.00	
≥ 30	5,465	12	0.17 (0.05–0.61)	3,635	22	0.33 (0.13–0.84)	0.405
	Low CDC ARIC DM risk scores < Median			High CDC ARIC DM risk scores ≥Median			
< 30	446	2	1.00	894	11	1.00	
≥ 30	4,076	5	0.05 (0.01–0.51)	5,024	29	0.35 (0.16–0.78)	0.058

^a.^ 25(OH)D conversions: 30 ng/mL = 75 nmol/L; 40 ng/mL = 100 nmol/L; 50 ng/mL = 125 nmol/L.

^b.^ Adjusted for sex, body mass index, waist circumference, calcium supplement intake and plasma levels of triglyceride and HDL-cholesterol

^c.^ *P*-heterogeneity between the two HRs in the two strata.

The inverse association of 25(OH)D with diabetes was stronger in individuals with hyperparathyroidism or who exercised regularly (*p*- heterogeneity = 0.006 and 0.046, respectively) (Table 5). Among those with hyperparathyroidism, those with 25(OH)D > 30 ng/ml had lower risk of diabetes (HR = 0.06, 95% CI 0.02–0.25). Among those reporting no exercise, those with 25(OH)D > 30 ng/ml also had lower risk (HR = 0.35, 95% CI 0.16–0.80). The association of 25(OH)D > 30 ng/ml with diabetes persisted despite metabolic syndrome or high NHANES III or CDC risk scores (HR = 0.42, 95% CI 0.17–1.00; HR = 0.33 95% CI 0.13–0.84; and HR = 0.39 95% CI = 0.18–0.85, respectively.

Plasma 1,25(OH)_2_D concentrations were not associated with incidence of diabetes or pre-diabetes (Table 3). Graphs are available from the authors.

## Discussion

Individuals with a 25(OH)D concentration > 30 ng/ml or 75 nmol/L had only one-third the incidence of diabetes as those with ≤ 30 ng/ml or 75 nmol/L. Those with a somewhat higher concentration of 25(OH)D > 50 ng/ml or 125 nmol/L had a much lower HR of 0.2.

The association of 25(OH)D with diabetes persisted after exclusion of participants who reported at baseline that they usually took vitamin D or calcium supplements. The inverse association of a higher 25(OH)D concentration ≥ 30 ng/ml or 75 nmol/L was consistent among individuals in higher traditional risk groups for diabetes such as those having metabolic syndrome or established diabetes risk factors according to standard scores for predicting risk of diabetes that are used by CDC and other organizations to predict incidence of diabetes. These include obesity and lack of regular exercise.

The finding of the present study that 25(OH)D concentration had a significantly inverse association with risk of diabetes is biologically plausible. Mice with the vitamin D receptor (VDR) null phenotype have higher incidence rates of diabetes [34], suggesting that the vitamin D pathway may be relevant to the pathogenesis of diabetes. Pancreatic beta cells have VDR, and vitamin D metabolites stimulate the pancreas to produce insulin [35].

Active metabolites of vitamin D also have been shown in animal models to protect pancreatic beta cells from cytokine-induced inflammation and apoptosis [34].

Only the 25(OH)D concentration was associated with lower risk of diabetes in the present study. One of the reasons may be the stability of 25(OH)D in circulation. 25(OH)D has a 75-fold longer half-life than 1,25(OH)_2_D [36]. Circulating 25(OH)D is also stable with respect to time, even in stored frozen plasma [37].

Although the number of cases of diabetes was much smaller than that of pre-diabetes, 25(OH)D levels were strongly inversely associated with risk of diabetes and weakly inversely associated with risk of pre-diabetes. This could be because pre-diabetes is a relatively mild condition, and includes many individuals who did not become diabetic.

Another possible reason is that people with pre-diabetes may be healthier due to better life-style behaviors. In our study, pre-diabetes participants were more likely to use vitamin D supplements and had higher HDL-cholesterol levels, lower triglyceride levels, BMI, and waist circumference, and were less likely to smoke cigarettes compared to diabetes patients.

The inverse association of 25(OH)D with diabetes was much stronger in individuals with hyperparathyroidism and who regularly exercised strenuously as shown in Table 5. Hyperparathyroidism, regardless of cause, is a common concern in the aged, particularly in women, due to renal deterioration, low estrogen, low calcium intake, and, on occasion, use of furosemide [38, 39]. It is also adversely associated with glucose tolerance and insulin resistance [40, 41].

In our cohort, individuals with hyperparathyroidism were likely to have higher risk for diabetes as shown in Table 5. Nevertheless, our finding that a higher 25(OH)D concentration has a beneficial effect on risk of diabetes is encouraging to older people. The beneficial effect of higher 25(OH)D could be due to improving insulin sensitivity and anti-inflammatory effects [40, 41[40,41] although the exact mechanisms by which a higher 25(OH)D concentration lowers diabetes risk remain unclear.

Individuals who were doing regular strenuous exercise were likely to have a lower HR associated with higher 25(OH)D concentration (Table 6). A similar association was present in those with higher levels of PTH (Table 6). Skeletal muscle has VDR [42]. Strenuous exercise itself has a favorable effect in controlling diabetes through increasing glucose utilization in muscle and insulin sensitivity [43].

### Limitations

The present study has several limitations. The study participants were relatively healthy middle to upper-middle class Caucasians, who had good access to health care. As a result, our findings may not be generalizable to other populations.

Rancho Bernardo, located 16 miles north of San Diego, CA, has sunny weather throughout the year. This natural environment helps the participants maintain higher 25(OH)D concentrations in blood without vitamin D supplementation. Previous studies have shown that 95% of 25(OH)D is a product of photosynthesis of vitamin D_3_ in the skin [44], and it is probable that there is more sunlight reaching the members of this community than members of previously studied communities.

Generalizability with certainty to areas less sunny than southern California could be examined by repeating the study. However, an association in the same direction as found in this study was present in another cohort whose members lived throughout the U.S. [45], although the association was not found in another cohort [46]. The present study will help resolve the question of whether the association is present in men and in older women.

It is also possible that the higher concentrations observed in this study might have been due to differences in laboratory assay techniques. The competitive binding protein assay may produce higher 25(OH)D results compared with radioimmunoassay and high-performance liquid chromatography [36, 47]. Concentrations of 25(OH)D and 1,25(OH)_2_D found in the Rancho Bernardo cohort may not be directly comparable to those in studies using different assays.

On the other hand, routine assays accurately rank individuals across the range of 25(OH)D levels [36], suggesting internal validity. Values for 25(OH)D were measured on a single blood specimen, but are known to have seasonal variation [48]; this may have weakened the observed association between these measures and incidence of diabetes. Nearly all other studies showing an association between 25(OH)D concentration and diabetes have also used a single measurement.

Comparison of the present study with a recent well-designed multi-center cohort study by Schafer et al. [20] of 25(OH)D and risk of diabetes in older adults is useful. The study by Schafer et al., in contrast to the present study, did not report the existence of an association of plasma 25(OH)D with risk of diabetes. It would be of value to try to explain the differences between that study and this one that might account for the differences in results.

Both studies used approximately the same well-established and highly respected cohort study design. The difference is unlikely to be merely a matter of a deficiency in basic study design. Both studies were performed by highly experienced research teams. Both studies ruled out the existence of diabetes at baseline. Both used well-regarded statistical methods, such as *t*-tests, chi-square tests and Cox proportional hazards regression. Both used either stratification and/or multiple regression to control for confounding.

There were more similarities between these studies than differences, but there were a few differences that may be instructive. One is that the study by Schafer et al. was conducted at 4 centers located mainly in the Northern and mid-Atlantic tiers of the US: Minneapolis, Pittsburgh, Baltimore, and Portland OR. These studies had a median latitude of 43 degrees N. This can be compared with the 33 degrees N latitude of Rancho Bernardo. Winter conditions can be harsh in these four areas, but winters in Rancho Bernardo are extremely mild. The mean 25(OH)D concentration was 23 ng/ml or 58 nmol/L in the Schafer et al. study, compared to 42 ng/ml or 105 nmol/L in the present study. It might be that the 25(OH)D concentrations in the subjects in the previous study tended to be in a range that is below the range in which 25(OH)D is inversely related to incidence of diabetes.

The present Rancho Bernardo cohort has the highest median 25(OH)D of any cohort study to date. This suggests a chance that there may be a threshold in the dose-response curve between 25(OH)D and incidence of diabetes. If a threshold for benefit from higher 25(OH)D exists, the present study suggests that it may be at about 30 ng/ml or 75 nmol/L. Thresholds are common features of dose-response curves [11]. Substantial numbers of subjects with serum 25(OH)D > 30 ng/ml or 75 nmol/L were not present in any cohorts previously studied, but were present in the Rancho Bernardo cohort.

Finally, an inverse association of 25(OH)D with risk of diabetes that was identified in the study by Schafer et al. lost its statistical significance after adjustment for BMI and other covariates. This adjustment is logical if BMI itself is the adverse factor. But if BMI happens to be a link in a possible causal chain from obesity to lower 25(OH)D to incidence of diabetes, the adjustment may have at least partially washed out the association that was found in the age- and clinic location-adjusted data in that study.

Based on the above contrasts between well-designed cohort studies such as that of Schafer et al. [20] and the present study, it is evident that more research is needed to delineate the contributory roles of BMI *per se* and of 25(OH)D to risk of diabetes. Such contributory roles may not be mutually exclusive, and the results of both studies could possibly be accurate. Meta-analyses may help provide context for understanding the diversity of findings of studies such as these [11, 13, 21, 22].

### Strengths

On the other hand, the present study has several strengths. The standard A. B. Hill criteria for causality in observational studies [49] were applied to the results of the present study, and the association of higher plasma 25(OH)D with lower risk of diabetes met most of the Hill criteria. The study was a prospective cohort study of individuals who were healthy volunteers when they enrolled, but developed diabetes during the study. The cohort study tends to have lower risk of reverse causation than a case-control study. Even when the hazard ratios were adjusted using regression and standard risk scoring systems for classical risk factors for diabetes, the findings persisted and remained similar in strength.

The results of this study suggest that targeting a plasma 25(OH)D concentration in the range of 50 ng/ml or 125 nmol/L might be useful in attempting to reduce the incidence rate of diabetes. However, it is thought by some workers that there may be an unknown degree of risk associated with maintaining 25(OH)D in this range. The main possible risk is one of hypercalcemia [50]. Another could be the chance of a higher risk of ischemic heart disease [50]. Results regarding whether such a risk exists have been mixed [50]. There have been no known reports of complications of vitamin D supplementation or high plasma 25(OH)D in our cohort.

There are still unresolved concerns about the desirable plasma target for 25(OH)D. At this moment, the authors would tentatively suggest that the target be no less than 40 ng/ml or 100 nmol/L. Other analysts considering the same data could reasonably choose other desired targets, such as 30 ng/ml or 75 nmol/L proposed by the Endocrine Society [51].

In conclusion, the higher plasma 25(OH)D concentrations of ≥ 50 ng/ml or 125 nmol/L in this cohort were independently associated with 80% lower incidence rates of diabetes. However, a target threshold of 50 ng/ml or 125 nmol/L is considerably above that recently recommended by an expert consensus panel that considered all known benefits and risks of vitamin D, and suggested instead a threshold of 30 ng/ml or 75 nmol/L [51]. As a result the possibility of a threshold higher than 30 ng/ml or 75 nmol/L should be approached with caution, pending replication of the findings [50].

Our study does not solve the basic question of whether individuals may need to seek vitamin D supplementation if needed to maintain a concentration of 30 ng/ml or 75 nmol/L, despite the possibility of any toxicity. A recent placebo-controlled randomized trial of a vitamin D weekly bolus supplement for pre-diabetes patients failed to prove a beneficial effect on 5-year incidence of diabetes [52]. Sufficient 25(OH)D levels obtained naturally from sunlight and food, not supplementation, might be more relevant than supplementation to reduce risk of diabetes. Larger cohort studies or long-term clinical trials would be desirable to help confirm whether this association is causal.

This study used a single measurement of 25(OH)D during a longitudinal study, since no other measurements were available. These measurements may have been more representative of serum levels during the 12.5 years of follow-up if two or more measurements had been made. The single measurement would have been of greatest concern if the study had found no association, since the absence of an association could have been due to use of a single measurement.

However an inverse association of 25(OH)D with incidence of diabetes was detected in this study despite the use of a single baseline measurement. It is possible that more associations could have been detected if there were more measurements of 25(OH)D during follow-up. On the other hand, 25(OH)D concentrations tend to be somewhat stable over time in adults [3]. The question of stability of season-specific 25(OH)D concentrations in adults over periods of 5 years or longer should be further studied in cohorts that have used multiple measurements during follow-up [3].

Both major metabolites of vitamin D were studied to determine whether an association was present for both. Only a low concentration of 25(OH)D is usually associated with diseases that are due to vitamin D-deficiency [1]. However the authors measured the 1,25(OH)_2_D concentration to confirm that the association was only for 25(OH)D and not present for 1,25(OH)_2_D. 1,25(OH)_2_D is tightly homeostatically regulated, and typically does not vary greatly among individuals [1, 29].

Serum 25(OH)D is generally accepted as the standard measure of vitamin D nutrition [1]. Most tissues can enzymatically convert it to 1,25(OH)_2_D [1]. In retrospect, measurement of 25(OH)D alone would have been adequate to test for an inverse association between vitamin D nutritional status and risk of diabetes. This study found no association of 1,25(OH)_2_D with risk of type 2 diabetes. We therefore do not suggest any benefit from measuring 1,25(OH)_2_D in future studies of the etiology of type 2 diabetes.

A decision was made in 1997 to include all members of the cohort who were alive. No sampling was done, so there was no design need for a formal sample size calculation. Lower than optimal power may have caused this study to miss the chance of detecting a true association, such as an inverse association between plasma 25(OH)D and risk of type 2 diabetes. However this association emerged as statistically significant. Still, the size of the present cohort may have been inadequately large to detect associations with other covariates. Therefore this study should not be used to rule out such associations.

Vitamin D supplementation only occurred in participants who were also taking calcium supplements. Therefore it was not practical to perform a separate analysis for vitamin D supplements.

## Supporting information

S1 Fig Flowchart of participants. (TIFF)

S1 Table Data, anonymized. (XLSX)
