# Supplementary figures and images for: Plasma 25-hydroxyvitamin D concentration and risk of type 2 diabetes and pre-diabetes: 12-year cohort study
Source: PLoS One. 2018 Apr 19;13(4):e0193070. doi: 10.1371/journal.pone.0193070 (PMC5908083; doi:10.1371/journal.pone.0193070)

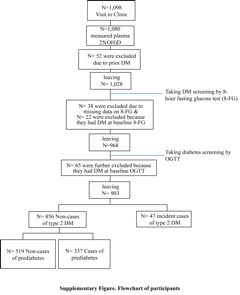

Supplement: S1 Fig — (TIFF) [file pone.0193070.s001.tiff]
